# Comparison of hypothesis- and data-driven asthma phenotypes in NHANES 2007–2012: the importance of comprehensive data availability

**DOI:** 10.1186/s13601-019-0258-7

**Published:** 2019-03-13

**Authors:** Rita Amaral, Ana M. Pereira, Tiago Jacinto, Andrei Malinovschi, Christer Janson, Kjell Alving, João A. Fonseca

**Affiliations:** 1 CINTESIS – Center for Health Technology and Services Research, Faculty of Medicine, University of Porto, Edifício Nascente, Piso 2, Rua Dr. Plácido da Costa, s/n, 4200-450 Porto, Portugal; 2 Department of Cardiovascular and Respiratory Sciences, Porto Health School, Porto, Portugal; 3 Department of Allergy, Instituto & Hospital CUF, Porto, Portugal; 4 Department of Medical Sciences, Clinical Physiology, Uppsala University, Uppsala, Sweden; 5 Department of Medical Sciences, Respiratory Medicine and Allergology, Uppsala University, Uppsala, Sweden; 6 Department of Women’s and Children’s Health, Paediatric Research, Uppsala University, Uppsala, Sweden; 7 MEDCIDS - Department of Community Medicine, Information, and Health Sciences, Faculty of Medicine, University of Porto, Porto, Portugal

**Keywords:** Asthma, Phenotypes, Population-based study, Unsupervised analysis

## Abstract

**Background:**

Half of the adults with current asthma among the US National Health and Nutrition Examination Survey (NHANES) participants could be classified in more than one hypothesis-driven phenotype. A data-driven approach applied to the same subjects may allow a more useful classification compared to the hypothesis-driven one.

**Aim:**

To compare previously defined hypothesis-driven with newly derived data-driven asthma phenotypes, identified by latent class analysis (LCA), in adults with current asthma from NHANES 2007–2012.

**Methods:**

Adults (≥ 18 years) with current asthma from the NHANES were included (n = 1059). LCA included variables commonly used to subdivide asthma. LCA models were derived independently according to age groups: < 40 and ≥ 40 years old.

**Results:**

Two data-driven phenotypes were identified among adults with current asthma, for both age groups. The proportions of the hypothesis-driven phenotypes were similar among the two data-driven phenotypes (p > 0.05). Class A < 40 years (*n* = 285; 75%) and Class A ≥ 40 years (n = 462; 73%), respectively, were characterized by a predominance of highly symptomatic asthma subjects with poor lung function, compared to Class B < 40 years (n = 94; 25%) and Class B ≥ 40 years (n = 170; 27%). Inflammatory biomarkers, smoking status, presence of obesity and hay fever did not markedly differ between the phenotypes.

**Conclusion:**

Both data- and hypothesis-driven approaches using clinical and physiological variables commonly used to characterize asthma are suboptimal to identify asthma phenotypes among adults from the general population. Further studies based on more comprehensive disease features are required to identify asthma phenotypes in population-based studies.

**Electronic supplementary material:**

The online version of this article (10.1186/s13601-019-0258-7) contains supplementary material, which is available to authorized users.

## Introduction

Airways diseases, such as asthma and chronic obstructive pulmonary disease (COPD), comprise a heterogeneous set of subtypes with different underlying pathophysiological mechanisms [1–3]. Both hypothesis-driven and data-driven methods can be used to classify patients into sub-groups of airways diseases [4–6].

The hypothesis-driven approach classifies airways diseases based on pre-defined criteria following immunopathology concepts and asthma literature, while in data-driven methods no prior disease classification is required [7, 8]. Data-driven approaches have provided insights into “novel” phenotypes of complex disease pathogenesis, suggesting disease stratification depending on the individual pathophysiologic characteristics [8–11].

Most studies on asthma phenotyping using data-driven methods emphasize patients with moderate to severe asthma and/or clinically-based settings [12–15]. Therefore, the generalization to the general asthma population may be limited.

Different types of data-driven methods have been widely used in airway diseases, such as hierarchical [12], partitioning [14], and latent class analysis (LCA) [10]. Notably, LCA appeared to account better for the heterogeneity of airways symptoms, compared to other commonly used data-driven approaches (e.g. partitioning around medoids) [16]. Moreover, the application of the latent class assignments developed from a national data source has previously demonstrated higher degrees of generalizability [17].

Recently, we reported a significant overlap between five distinct hypothesis-driven asthma phenotypes in adults from the general population included in the US National Health and Nutrition Examination Survey (NHANES) [18]. We have emphasized that a combination of clinical information and biomarkers, using a more comprehensive data analysis approach, such as data-driven methods, could provide a better taxonomy of non-severe asthma.

In this study, we aimed to compare previously defined hypothesis-driven asthma phenotypes [18] with data-driven asthma phenotypes derived by applying LCA to a sample of adults representative of the US general population.

## Methods

### Study setting and participants

We have included subjects that participated in the NHANES study, a nationally representative survey of the civilian, non-institutionalized US population performed with the aim of gathering data regarding health and nutritional status. Protocols were approved by the National Center for Health Statistics Research Ethics Review Board and all participants gave written informed consent. Detailed information can be found in the NHANES documentation (www.cdc.gov/nchs/nhanes.htm).

Data from three NHANES surveys was used (n = 30,442). We included adults (≥ 18 years old) with current asthma (n = 1059), defined by a positive answer to the questions [18]: “Has a doctor ever told you that you have asthma?” together with “Do you still have asthma?”, and either “wheezing/whistling in the chest in the past 12 months” or “asthma attack in the past 12 months.”

### Variables

Anthropometric and demographic characteristics, such as age, gender, body mass index (BMI), and smoking status were analysed, as well as blood eosinophils (B-Eos) count, fraction of exhaled nitric oxide (FeNO) and spirometric parameters. FeNO and spirometry were performed following ATS/ERS recommendations [19, 20]. Basal predicted values of forced expiratory volume during the first second (FEV_1_) and forced vital capacity (FVC) were calculated [21, 22] and abnormal values were defined as being below the lower limit of normal (LLN) [23].

### Hypothesis-driven asthma phenotypes

The analysis based on the report of smoking status, presence of obesity and inflammatory markers enabled the definition of five asthma phenotypes [18]: B-Eos-high asthma phenotype, if B-Eos ≥ 300/mm^3^; FeNO-high asthma, if FeNO ≥ 35 ppb; B-Eos&FeNO-low asthma, if B-Eos < 150/mm^3^ and FeNO < 20 ppb; asthma with obesity (AwObesity), if BMI ≥ 30 kg/m^2^; and asthma with concurrent COPD (AwCOPD), if subjects had self-reported chronic bronchitis/emphysema with age of diagnosis ≥ 40 years and being either a current or an ex-smoker (ever smoked). Subjects were considered as “non-classified” if they did not meet the criteria for any of the defined asthma phenotypes. Additionally, to account for individuals with probable co-existence of asthma and COPD and minimize age as a confounding variable, we conducted the analysis considering two age groups: < 40 and ≥ 40 years old [18].

### Data-driven asthma phenotypes

LCA was used to identify asthma phenotypes in an unsupervised manner (data-driven approach). Two models for “current asthma” were developed (Additional file 1: Table S1): Model 1 was based on the 4 variables previously used to define the hypothesis-driven asthma phenotypes (BMI ≥ 30 kg/m^2^, ever-smoking status, FeNO ≥ 35 ppb, B-Eos ≥ 300/mm^3^) [18]; and in Model 2, we have added to the former 4 variables, sex, early asthma onset (< 16 years old), wheezing-related questions (presence/absence of at least one wheezing attack, wheezing with exercise, sleep disturbance by wheezing, limit activity by wheezing, absenteeism by wheezing), asthma-related emergency department (ED) visit in the previous 12 months, FEV_1_/FVC < LLN, FEV_1_ < LLN, and self-reported hay fever.

Additionally, to explore the results in different “asthma populations”, we’ve developed two other models using similar variables. For the “ever asthma” subgroup (model 3) we included subjects with a positive answer to “Has a doctor ever told you that you have asthma?” (n = 2611); and for the “difficult asthma” (model 4) we included subjects with poor asthma-related outcomes, defined as current asthma plus, at least, one of the following: asthma-related ED visit, FEV_1_ < LLN, or oral corticosteroids use in the past 30 days (n = 673) (Additional file 1: Table S1).

Latent class models were derived independently for each age group, using the same variables, and a secondary analysis without stratifying by age was done on the three asthma subgroups. The most appropriate number of clusters was determined by examining commonly used criteria [24]. Further methodological details are found in the Additional file 1.

### Statistical analysis

All analyses considered the complex multistage sampling and 6-year sampling weights provided by the NHANES documentation [25]. LCA was performed with MPlus (version 6.12), that considered the complex survey design of NHANES when performing LCA-modelling. All other analysis was performed in Stata/IC 15.1 (Stata Corp, College Station, TX, USA). A p-value < 0.05 was considered statistically significant.

## Results

We included 1059 adults with current asthma. The weighted proportions of the previously defined hypothesis-driven asthma phenotypes, according to age groups (< 40 and ≥ 40 years old) were, respectively: 42% and 53% with AwObesity; 34% and 37% with B-Eos-high asthma; 26% and 21% for B-Eos&FeNO-low; 18% and 19% with FeNO-high asthma; and 19% AwCOPD, in the older group [18]. In addition, 17% and 12% of the individuals in the < 40 and ≥ 40 years old groups, respectively, were categorized as “non-classified”.

In Model 1, LCA was not able to differentiate any asthma subgroup among subjects with current asthma (Additional file 1: Table S1). On the other hand, by adding more asthma-related variables (Model 2), LCA identified a two-class model as the best solution for both age groups (Table 1, Additional file 1: Table S1). Classes A < 40 years (n = 290; 75%) and A ≥ 40 years (n = 494; 73%) had marked predominance of highly symptomatic asthma subjects, with poorer lung function, compared to classes B < 40 years (n = 96; 25%) and B ≥ 40 years (n = 179; 27%), respectively (Table 1). Regarding inflammatory markers, the proportion of patients with high levels of B-Eos and FeNO was not significantly different between classes, both in the younger group (p = 0.99 and p = 0.82, respectively) and in the older group (p = 0.57 and p = 0.53).

**Table 1:**
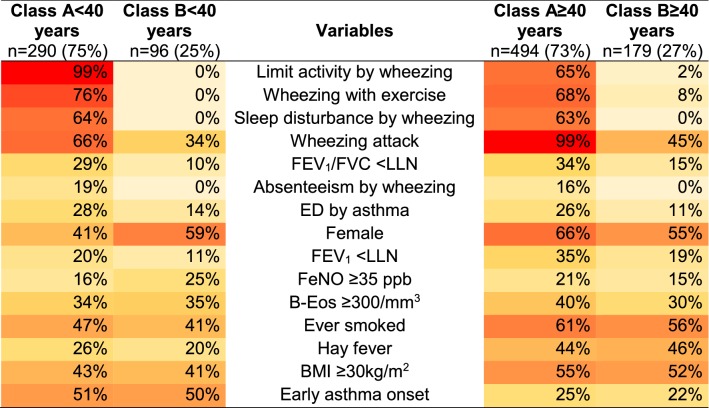
Proportions of each variable according to the LCA-classes identified in Model 2 (subjects with current asthma, n = 1059)

*FEV*_1_ forced expiratory volume in the first second, *FVC* forced vital capacity, *LLN* lower limit of normality, *ED* emergency department, *FeNO* fractional exhaled nitric oxide, *B-Eos* blood eosinophils count, *BMI* body mass index

Variables are ordered by the highest mean difference between the 2 classes of each age group and each coloured box represents the prevalence of the variables within the class, ranging from 0% (light yellow) to 100% (red)

**Fig. 1:**
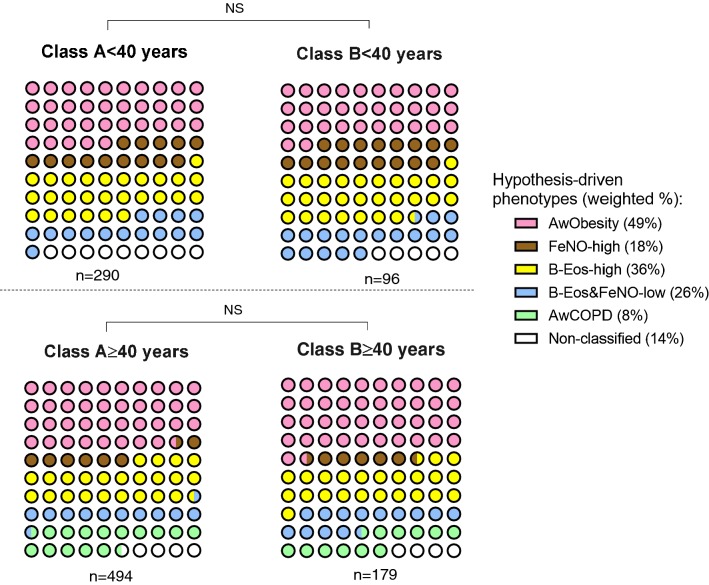
Distribution of the hypothesis-driven asthma phenotypes according to the data-driven classes identified in Model 2. Both Class A < 40 and Class A ≥ 40 are the phenotypes with more asthma-related symptoms and low lung function. No significant differences (p > 0.05) were observed between the proportions of the hypothesis-driven within the data-driven phenotypes. *NS* non-significant

Figure 1 shows that the distribution of the hypothesis-driven phenotypes is similar (p > 0.05) in both classes identified by LCA regardless age group.

Additionally, LCA identified 2 classes on the models for “ever-asthma” and “current asthma” without stratifying by age, but not for the difficult-asthma sub analysis where no subgroup was identified (Additional file 1: Table S1).

## Discussion

This was the first study comparing previously defined hypothesis-driven asthma phenotypes with data-driven ones in a sample representative of the US general population. The proportions of the hypothesis-driven phenotypes were similar between the two data-driven phenotypes obtained by LCA using clinical and physiological variables commonly used to characterize asthma.

Previous studies using data-driven approaches contributed to the definition of clusters/phenotypes based on similarities in clinical and inflammatory biomarkers [9, 12–14]. However, these approaches have been scarcely applied to adults with asthma from population-based studies. The studies from Siroux et al. [26] and Mäkikyrö et al. [27] provided further evidence for identifying subgroups of asthma based on clinical markers and questionnaire data commonly available in primary health care or large epidemiological studies and found a larger range of asthma phenotypes.

Our study showed that performing LCA with the variables used to define some of the most common hypothesis-driven asthma phenotypes, could not identify subgroups within adults with current asthma from the general population. By including additional clinical and physiological variables commonly used to classify asthma, LCA identified two data-driven phenotypes in the same subjects. Overall, these phenotypes only differed in symptom frequency and lung function parameters. Inflammatory biomarkers, presence of obesity, smoking status, age of asthma onset and self-reported hay fever were not different between classes.

Moreover, using a less stringent asthma definition (ever asthma) and in subjects with poor clinical outcomes (difficult asthma), these variables were also suboptimal to differentiate asthma subgroups.

In contrast to studies with severe asthma patients, our results suggest that, for the general asthma population, the clinical and physiological variables available to classify asthma and commonly used predefined cut-offs seem to be insufficient to identify specific phenotypes. The inclusion in data-driven models of additional easily measurable biomarkers that have already been shown to be helpful in discriminating asthma phenotypes in this population (e.g. serum IgE and/or periostin) [28, 29], combined with comprehensive clinical, physiologic, and/or disease features, might result in the identification of more precise phenotypes. Also, the identification of new, more accurate biomarkers could also improve phenotyping [30]. Furthermore, the use of fixed cut-offs values, although common and more intuitive for daily clinical practice, may potentially miss more complex, and yet unidentified phenotypes. The use of absolute values (as seen in other studies [13, 31, 32]), or appropriate reference equations for predicted values [33, 34] could be more adequate.

Similarly, research efforts are being made to integrate clinical characteristics with available biomarkers to identify data-driven asthma phenotypes in children [35, 36]. However, the obtained phenotypes vary on key features that are more pronounced during childhood, including natural history of wheeze over time [37], suggesting that further work is required to compare data- and hypothesis-driven approaches to identify asthma phenotypes in children.

Limitations inherent to a survey study design must be acknowledged and the self-reported variables may lead to misclassifications and information biases; to account for these biases, we used previously validated definitions [38, 39]. Also, despite including the most commonly used variables for respiratory disease assessment available in the NHANES study, when using the less stringent asthma definition, the differentiation of asthma subgroups was not improved in this population. However, to reduce the risk of poor LCA-class differentiation, we did not include any of the variables used in the asthma groups definition into the LCA models. Finally, LCA modelling should comprehend all the domains relevant to the understanding of the disease to classify observations into discrete and mutually exclusive classes [40], suggesting that the use of predefined cut-offs and the lack of data regarding, for example, objective assessment of atopy, nasal and ocular symptoms (which have proved to be useful in the stratification of allergic respiratory diseases [10, 41]), may have limited the ability to differentiate specific asthma phenotypes using unsupervised analysis.

In conclusion, this brief communication extends our previous work on the need for a broader data analysis combining different asthma-related domains for differentiating phenotypes in the general asthma population [18]. The clinical and physiological variables commonly used to subdivide asthma seem to be insufficient to differentiate specific asthma phenotypes among adults from the general population, irrespective of using data-driven or hypothesis-driven approaches. Further studies based on more comprehensive disease features are required to identify asthma phenotypes with the potential to be useful for clinicians and for population-based research.

## Additional file

**Additional file 1.** Supplementary methods.

## Abbreviations

ATS/ERS: American Thoracic Society/European Respiratory Society
AwCOPD: asthma with concurrent COPD
AwObesity: asthma with obesity
B-Eos: blood eosinophils
BMI: body mass index
COPD: chronic obstructive pulmonary disease
ED: emergency department
FeNO: fraction of exhaled nitric oxide
FEV_1_: forced expiratory volume during the first second
FVC: forced vital capacity
LCA: latent class analysis
LLN: lower limit of normal
NHANES: National Health and Nutrition Examination Survey
US: United States
