# Supplementary material for: Comparison of hypothesis- and data-driven asthma phenotypes in NHANES 2007–2012: the importance of comprehensive data availability
Source: Clin Transl Allergy. 2019 Mar 13;9:17. doi: 10.1186/s13601-019-0258-7 (PMC6419396; doi:10.1186/s13601-019-0258-7)
Supplement: Supplementary file 1 — Additional file 1. Supplementary methods. [file 13601_2019_258_MOESM1_ESM.doc]

# Online Supplement: Brief communication

# **Comparison of hypothesis- and data-driven asthma phenotypes in NHANES 2007-2012: the importance of comprehensive data availability**

Rita Amaral, MSc. ^(1,2)^, Ana M. Pereira, M.D. ^(1,3)^, Tiago Jacinto, Ph.D. ^(1,2)^, Andrei Malinovschi, M.D., Ph.D. ^(4)^, Christer Janson, M.D., Ph.D. ^(5)^, Kjell Alving, M.D., Ph.D. ^(6)^, João A. Fonseca, M.D., Ph.D. ^(1,3,7)^

# Methods

### **NHANES study design**

The NHANES is a nationally representative survey of the civilian, non-institutionalized U.S. population with the aim of gathering data regarding health and nutritional status [1]. In each survey, participants were selected using a complex stratified multistage cluster sampling strategy and it combines interviews and physical examinations. The survey examines a nationally representative sample of about 5,000 persons each year. These persons are located in counties across the US [1].

### **Variables definition**

The main questions used to define the asthma-related questions were collected from the structured clinical interview assessment. Variables were defined by the following questions:

- *Smoking status*: current smoker defined as smoking at least one cigarette every day for one year; ex-smokers reported having quit smoking for more than one month; non-smokers reported neither smoking nor ex-smoking.

- *Self-reported chronic bronchitis/emphysema*: Positive answer to either “Has a doctor ever told you/SP that you had chronic bronchitis?” or “Has a doctor ever told you/SP that you had emphysema?”

- *Age of chronic bronchitis/emphysema diagnosis*: defined with the answer to the question “How old were you when you were first told you had chronic bronchitis/emphysema?”

- *Age of asthma onset*: defined with the answer to the question “How old were you when you were first told you had asthma?”

- *Wheezing attack*: defined if at least one was reported to the question “In the past 12 months, how many attacks of wheezing or whistling have you?”

- *Wheezing with exercise*: Positive answer to “In the past 12 months, has your chest sounded wheezy during or after exercise or physical activity?”

- *Sleep disturbance by wheezing*: defined if at least one was reported to the question “In the past 12 months, how often, on average, has your sleep been disturbed because of wheezing?”

- *Limit activity by wheezing*: defined if at least a little amount was reported to the question to “During the past 12 months, how much did you/SP limit your usual activities due to wheezing or whistling?”

- *Absenteeism by wheezing*: defined if at least 1 to 7 days option was reported to the question “During the past 12 months, how many days of work or school did you miss due to wheezing or whistling?”

- A*sthma-related emergency department visits*: Positive answer to “During the past 12 months, have you had to visit an emergency room or urgent care center because of asthma?”

*- Hay fever*: Positive answer to “During the past 12 months, have you had an episode of hay fever?”

- *Oral corticosteroids use:* Positively answer to “In the past month, have you used or taken medication for which a prescription is needed?” and then reported OCS use. [2]. Information regarding the use of oral corticosteroids was analyzed.

**Data collection**

Examinations and interviews are conducted in a mobile examination center (MEC), which is composed of specially equipped trailers.

Respiratory health was collected by means of an assessment of lung function testing, or spirometry, and a measurement of fractional exhaled nitric oxide (FeNO). Spirometric procedures followed the ATS/ERS recommendations [3], and a minimum of 3 acceptable and reproducible measurements were obtained (detailed procedures in: www.cdc.gov/nchs/data/nhanes/nhanes_11_12/Spirometry_Procedure_Manual.pdf). Predicted values of basal FEV_1_ and FEV_1_/FVC were calculated according to the method of Hankinson *et al*. [4], with a correction factor for ethnicity [5]. Abnormal values were defined as follows: FEV_1_ and FEV_1_/FVC less than the lower limit of normal (LLN) [6]. FeNO measurements were performed using the analyzer NIOX MINO® (Aerocrine, Solna, Sweden) [1], and values not fulfilling ATS/ERS criteria were excluded (n=285) [7].

The laboratory component includes the collection and processing of various biological

and environmental specimens including blood and urine. Blood eosinophils (B-Eos) were part of the complete blood counts and assessed on the Beckman Coulter MAXM® instrument (Beckman Coulter, Fullerton, Calif). A detailed description of the laboratory method used can be found elsewhere [8].

### **LCA methodology**

Latent class analysis (LCA) was applied to identify the phenotypes without the need for historical or a priori assumptions. LCA is a model-based approach used to identify underlying unobserved (latent), mutually exclusive subgroups (classes) based on categorical manifest variables [9]. Each participant was assigned to one class for each measure with the highest posterior class membership probability. The most appropriate number of classes was determined by examining commonly used criteria, including the Akaike Information Criterion (AIC), the Bayesian Information Criterion (BIC), the sample size-adjusted BIC, the Lo-Mendell-Rubin Likelihood Ratio Test, and the entropy [10].

### **Statistical analysis**

In accordance with the NHANES sampling design, the full sample 2-year mobile examination center weight was used to obtain weighted percentages adjusted to the US adult population.

Categorical variables were described as frequencies and weighted percentages. Chi-square tests and a Mann-Whitney U-test were used to compare classes for categorical and continuous variables, respectively.

# References

1. CDC. Respiratory Health ENO Procedures Manual. In: National Health and Nutrition Examination Survey. 2011. <http://www.cdc.gov/nchs/data/nhanes/nhanes_11_12/Respiratory_Health_ENO_Procedures_Manual.pdf>. Accessed 9 Dec 2018

2. Prescription Medications - Drug Information. <http://wwwn.cdc.gov/Nchs/Nhanes/1999-2000/RXQ_DRUG.htm>. Accessed 15 Oct 2018

3. Miller MR. Standardisation of spirometry. Eur Respir J. 2005;26(2):319–38.

4. Hankinson JL, Odencrantz JR, Fedan KB. Spirometric Reference Values from a Sample of the General U.S. Population. Am J Respir Crit Care Med. 1999;159(1):179–87.

5. Hankinson JL, Kawut SM, Shahar E, Smith LJ, Stukovsky KH, Barr RG. Performance of American Thoracic Society-Recommended Spirometry Reference Values in a Multiethnic Sample of Adults. Chest. 2010;137(1):138–45.

6. Stanojevic S, Wade A, Stocks J, Hankinson J, Coates AL, Pan H, et al. Reference Ranges for Spirometry Across All Ages. Am J Respir Crit Care Med. 2008;177(3):253–60.

7. Silkoff PE. ATS/ERS Recommendations for Standardized Procedures for the Online and Offline Measurement of Exhaled Lower Respiratory Nitric Oxide and Nasal Nitric Oxide, 2005. Am J Respir Crit Care Med. 2005;171(8):912–30.

8. CDC. Complete Blood Count. In: Laboratory Procedure Manual. 2013. <http://www.cdc.gov/nchs/data/nhanes/nhanes_11_12/cbc_g_met_he.pdf>. Accessed 9 Dec 2018

9. Wang J, Wang X. Structural Equation Modeling: Applications Using Mplus. West Sussex: Wiley; 2012.

10. Han J, Kamber M, Pei J. Data Mining: Concepts and Techniques. 3rd ed. Waltham: Morgan Kaufmann Publishers; 2012.

# Table S1

**Description of the LCA-models and the respective included variables in the different “asthma populations”.** In gray is the selected model used to compare with the hypothesis-driven asthma phenotypes.

|  | **Variables included in the model** | **Number of LCA classes**  (p-value*) | | |
| --- | --- | --- | --- | --- |
|  |  | <40 years old | ≥40 years old | Without stratifying by age |
| **Model 1**  **“Current asthma”** †  n= 1,059 | - BMI ≥30kg/m^2^ (Y/N)  - Ever smoked (Y/N)  - FeNO ≥35ppb (Y/N)  - B-Eos ≥300/mm^3^ (Y/N) | 1  (N/A) | 1  (N/A) | 1  (N/A) |
| **Model 2**  **“Current asthma”** †  n= 1,059 | - BMI ≥30kg/m^2^ (Y/N)  - Ever smoked (Y/N)  - FeNO ≥35ppb (Y/N)  - B-Eos ≥300/mm^3^ (Y/N)  - Sex (M/F)  - Early asthma onset (Y/N)  - Wheezing-related questions (Y/N): at least one wheezing attack, wheezing with exercise, sleep disturbance/limit activity/absenteeism by wheezing  - FEV_1_/FVC <LLN (Y/N)  - FEV_1_<LLN (Y/N)  - Asthma-related ED visit (Y/N)  - Hay fever (Y/N) | 2  (0.003) | 2  (0.04) | 2  (<0.001) |
| **Model 3**  **“Ever asthma”** ‡  n= 2,611 | - BMI ≥30kg/m^2^ (Y/N)  - Ever smoked (Y/N)  - FeNO ≥35ppb (Y/N)  - B-Eos ≥300/mm^3^ (Y/N)  - Sex (M/F)  - Early asthma onset (Y/N)  - Wheezing-related questions (Y/N): at least one wheezing attack, wheezing with exercise, sleep disturbance/limit activity/absenteeism by wheezing  - FEV_1_/FVC <LLN (Y/N)  - FEV_1_<LLN (Y/N)  - Asthma-related ED visit (Y/N)  - Hay fever (Y/N) | 2  (<0.001) | 2  (0.001) | 2  (<0.001) |
| **Model 4**  **“Difficult asthma”** ¥  n= 673 | - BMI ≥30kg/m^2^ (Y/N)  - Ever-smoking status (Y/N)  - FeNO ≥35ppb (Y/N)  - B-Eos ≥300/mm^3^ (Y/N)  - Sex (M/F)  - Early asthma onset (Y/N)  - Wheezing-related questions (Y/N): at least one wheezing attack, wheezing with exercise, sleep disturbance/limit activity/absenteeism by wheezing;  - Hay fever (Y/N) | 1  (N/A) | 1  (N/A) | 1  (N/A) |

N/A: not applicable; Y/N: Yes/No; BMI: body mass index; FeNO: Fractional exhaled nitric oxide; B-Eos: Blood eosinophils count; FEV1: Forced Expiratory Volume in the first second; FVC: Forced vital capacity; LLN: Lower limit of normality; ED: Emergency department.

* p-value obtained by the Lo-Mendell-Rubin Likelihood Ratio Test.

† Current asthma defined as a positive answer to the questions: “Has a doctor ever told you that you have asthma?” together with “Do you still have asthma?”, and either “wheezing/whistling in the chest in the past 12 months” or “asthma attack in the past 12 months.”

‡ Ever asthma defined as a positive answer to “Have you ever had asthma?”

¥ Difficult asthma defined as current asthma plus, at least, one of the following: asthma-related ED visit in the previous 12 months, FEV1<LLN, or LLN, or oral corticosteroids use in the past 30 days
